# Anti-inflammatory effect of biologic therapy in patients with psoriatic disease: A prospective cohort FDG PET study

**DOI:** 10.1007/s12350-023-03204-8

**Published:** 2023-02-08

**Authors:** Kevin E. Boczar, Rob S. Beanlands, Steven J. Glassman, Jerry Wang, Wanzhen Zeng, Robert A. deKemp, Natalie C. Ward, Christophe A. Fehlmann, George A. Wells, Jacob Karsh, Girish Dwivedi

**Affiliations:** 1 University of Ottawa Heart Institute, Ottawa, ON Canada; 2 School of Epidemiology and Public Health, University of Ottawa, Ottawa, ON Canada; 3 University of Ottawa, Ottawa, ON Canada; 4 Division of Cardiology, Department of Medicine, Perelman School of Medicine of the University of Pennsylvania, Philadelphia, PA USA; 5 Division of Dermatology, The Ottawa Hospital, Ottawa, ON Canada; 6 School of Medicine, University of Western Australia, Perth, WA Australia; 7 The Ottawa Hospital Research Institute, Ottawa, ON Canada; 8 Division of Emergency Medicine, Geneva University Hospitals, Geneva, Switzerland; 9 Research Methods Centre, University of Ottawa Heart Institute, Ottawa, ON Canada; 10 Division of Rheumatology, The Ottawa Hospital, Ottawa, ON Canada; 11 Department of Advanced Clinical and Translational Cardiovascular Imaging, Harry Perkins Institute of Medical Research, Murdoch, Australia; 12 Department of Cardiology, Fiona Stanley Hospital, Murdoch, WA Australia; 13 School of Biomedical Sciences, Curtin University, Bentley, WA Australia

**Keywords:** Psoriatic arthritis, inflammation, PET imaging, cardiovascular disease

## Abstract

**Aim:**

The aim of the study was to evaluate the changes in central vascular inflammation measured by FDG PET and myocardial blood flow reserve (MFR) determined by ^82^Rb PET following therapy with biologic agents for 6 months in patients with psoriatic arthritis (PsA) and/or cutaneous psoriasis (PsO) (group 1) and compare with PsO subjects receiving non-biologic therapy (group 2) and controls (group 3).

**Methods and Results:**

Target-to-background ratio (TBR) by FDG PET in the most diseased segment of the ascending aorta (TBR_max_) was measured to assess vascular inflammation. ^82^Rb PET studies were used to assess changes in left ventricular MFR. A total of 34 participants were enrolled in the study (11 in group 1, 13 in group 2, and 10 controls). A significant drop in the thoracic aorta uptake was observed in the biologic-treated group (ΔTBR_max_: − .46 ± .55) compared to the PsO group treated with non-biologic therapy (ΔTBR_max_: .23 ± .67). Those showing response to biologic agents maintained MFR compared to who showed no response.

**Conclusion:**

In a cohort of psoriasis patients treated with biologics, FDG uptake in the thoracic aorta decreased over the study period. Patients who demonstrated a significant anti-inflammatory response on FDG PET imaging maintained their MFR compared to non-responders.

## Introduction

Psoriatic arthritis (PsA) is a disease of chronic inflammation and occurs in 14-30% of patients with skin or nail psoriasis (PsO).^1,2^ Similar to other diseases of chronic inflammation, patients with PsA or PsO have higher than expected rates of cardiovascular (CV) disease and an increased risk of major adverse cardiovascular events (MACE).^3,4^ Indeed, CV disease is the single leading cause of death in patients with PsA and dermatologic psoriasis and may account for > 50% of all deaths in these populations.^5^

Systemic inflammation is thought to be the key mediator in the link between psoriasis and CV disease.^6^ Patients with psoriasis are in a state of chronic immune activation and have increased levels of inflammatory cytokines, such as tumor necrosis factor (TNF)-α.^7^ Interestingly, data have linked psoriasis severity with the degree of systemic inflammation.^8^ Chronic inflammation has a negative effect on the vasculature and plays a pivotal role in the mediation of the peripheral microvascular dysfunction that is seen in patients with psoriasis.^9^ A better understanding of the pathophysiology and potential treatment options behind the accelerated atherosclerosis and inflammation that is seen in patients with psoriasis remains a priority.

F-18-fluorodeoxyglucose positron emission tomography (FDG PET) provides an attractive and pragmatic means to measure and follow vascular inflammation in these patients, as it is the most widely validated and applied imaging probe in this regard.^10–12^ Previous studies have shown that its uptake predicts MACE;^13–15^ and it is also highly sensitive to short-term treatment effects.^16,17^ Furthermore, PET myocardial perfusion imaging in conjunction with rubidium-82 (^82^Rb) affords the ability to assess regional myocardial blood flow to the left ventricle in absolute terms (mL·min^−1^·g^−1^).^18^ Rubidium-82 PET has been established as a nuclear imaging method for accurate, reproducible, and routine quantification of myocardial blood flow and myocardial blood flow reserve [MFR (stress/rest perfusion)] in humans and in animal models of disease.^19–22^

Patients with psoriasis, especially those requiring systemic biologic therapy, have increased vascular inflammation.^8^ We hypothesized that it is increased central vascular inflammation in these patients that is contributing to the development of accelerated atherosclerosis. We sought to use FDG PET to quantify vascular inflammation in the ascending aorta in patients with PsA and PsO and hypothesized that thoracic aortic vascular inflammation would improve in these patients following therapy with biologic agents compared to psoriasis patients receiving non-systemic therapies and control patients with non-inflammatory skin/joint disease. Secondly, we sought to explore changes in MFR over time after treatment with biologic agents and explore the relationship between changes in vascular inflammation, as measured by FDG PET, to changes in MFR determined by ^82^Rb PET.

## Methods

This was a prospective cohort clinical study designed to determine the effect of biologic therapy on vascular inflammation and MFR in patients with PsA compared to cohorts not treated with biologic therapies. Study approval was obtained from the University of Ottawa Heart Institute’s Research Ethics Board in accordance with the principles of Declaration of Helsinki. All study participants provided written informed consent.

We studied 3 patient groups which were consecutively enrolled: (i) patients with PsA and/or PsO who were to be started on anti-TNF-α, anti-interleukin (IL)-17, or anti-IL-12/23 therapy biologic agents, (ii) patients with psoriasis managed on non-biologic therapies, and (iii) control patients with non-inflammatory skin or joint conditions. Participants were enrolled from the Rheumatology and Dermatology Clinics at the Ottawa Hospital, Ottawa, Ontario, Canada. A convenience sample was used, and all participants underwent ^82^Rb PET and FDG PET examinations at baseline and at 6 months. Demographic and anthropometric data including age, sex, ethnicity, height, weight, waist circumference, and blood pressure were recorded as well as current medications, medical history, smoking status, and family history of CV disease.

Group 1 consisted of subjects with PsA and/or PsO scheduled for initiation of anti-TNF-α therapy, anti-IL-17, or anti-IL-12/23 therapy with prior failed response to traditional disease-modifying anti-rheumatic drugs (DMARDs). After inclusion in the study, subjects in Group 1 received 6-month treatment with a biologic therapy following the baseline study investigations. Patients with PsA were included if they met the classification criteria for PsA^21^ and had persistent joint inflammation measured as 5 or more swollen joints, despite therapy for at least three months, each with 2 or 3 conventional DMARDs (methotrexate, leflunomide, or sulfasalazine). To minimize the effect that duration of the disease could play on the results, only patients with a recent diagnosis of PsA were included (i.e., patients that had a diagnosis of PsA for > 3 months and < 2 years).^23^ The treatment method chosen for patients was decided by the patient’s treating rheumatologist.

Group 2 included subjects with psoriasis on non-biologic treatment (i.e., topical medications, acitretin, or phototherapy only). Psoriasis Area and Severity Index (PASI) score was used for the assessment of severity of dermatological psoriasis.^24^

Group 3 included patients with an established diagnosis of non-inflammatory joint diseases, such as osteoarthritis. These control patients were identified by a detailed history and physical examination, with normal baseline blood tests (full blood count, renal function, fasting glucose, and lipid profile).

### Primary outcome: FDG PET imaging of ascending aortic inflammation

The primary outcome of our study was the change in vascular inflammation in the ascending aorta, which was measured according to standardized methods^16^ as the target-to-background ratio (TBR) in the most diseased segment of the ascending aorta at baseline compared to 6-month follow-up (TBR_max_). All patients underwent FDG PET with low-dose computed tomography at baseline and then following 6-month follow-up. Patients in group 1 started on biologic agents following their baseline FDG PET. FDG PET images were analyzed to determine TBR_max_ values by utilizing previously published and validated methodology.^16^ In short, we first obtained the maximum standardized uptake value (SUV) for the most diseased segment of the ascending aorta by averaging the SUV for 3 consecutive axial slices centered on the highest uptake slice and the adjacent slices superior and inferior to it, providing approximately 1 cm of the most inflamed section of the aortic wall. We then calculated the TBR_max_ by determining the ratio of this SUV_max_ for the most inflamed region of the ascending aorta to background venous activity, derived from an image region in the superior vena cava. On the follow-up scan in the treatment group, the same 3 slice locations were used to calculate the follow-up TBR_max_. Of note, the FDG PET analysis was performed by an independent reader blinded to clinical details.

### Secondary outcome: FDG PET imaging of other aortic inflammation

As a secondary analysis, we also evaluated the change in vascular inflammation in other areas of the aorta, including the aortic arch and descending aorta. Similar to the methods outlined above for the ascending aorta, we measured this as the TBR in the most diseased segment of the respective portion of the aorta (arch or descending aorta) at baseline compared to 6-month follow-up (TBR_max_).

### Secondary outcome: ^82^Rb PET for MFR

Dynamic ^82^Rb PET was performed according to previously published and validated methodology.^25,26^ In short, absolute myocardial blood flow was calculated at rest and during dipyridamole stress using our FlowQuant© analysis software. The ^82^Rb analysis was performed by an independent reader blinded to clinical details.

### Post hoc analysis: MFR

To better understand the relationship of changes in vascular inflammation to microvascular function, in a post hoc analysis we evaluated the change in MFR values in the subgroup of PsA patients treated with biologic agents. We compared the MFR of patients in the biologic group that had a response in their TBR_max_ (that was greater or equal to the median delta TBR_max_ of this group) to those that had a response that was less than the median (i.e., post hoc analysis for effect modification).

### Statistical methods

Descriptive statistics (such as median with interquartile range (IQR) for continuous variables, frequencies with percentages for categorical variables) are used to summarize the three study groups’ baseline demographic and clinical variables. Continuous variables are presented as medians with IQR and categorical variables are presented as frequencies and percentages (unless otherwise stated). Demographic characteristics are presented as median (IQR) or percentage as appropriate. Differences between patient groups were evaluated by the Fisher exact test for discrete clinical variables and by the Kruskal–Wallis test for continuous variables. Follow-up data were collected as scheduled. All the tests were two-sided and a *P* value of < .05 was considered statistically significant. Within-group testing of changes in baseline and 6-month TBR_max_ and left ventricle (LV) MFR values were conducted with Wilcoxon Signed Rank testing. The relative changes in TBR_max_ and LV MFR over the study period were compared between groups using a generalized linear mixed effects model (GLMM) for repeated measures analysis. Variables included in the model were age, body mass index, sex, and baseline aortic TBR_max_. Missing data were considered missing at random and complete case analysis was used to handle the missingness. We also performed multiple imputation as a sensitivity analysis to assess the effect of missingness on our results using a GLMM with baseline and follow-up TBR_max_ included in the model (thereby adjusting for differences in baseline aortic inflammation across groups). Statistical analyses were performed using MedCalc for Windows version 12.0 (MedCalc Software, Ostend, Belgium) and Stata/IC for Windows version 16.1 (StataCorp LLC, TX, USA).

## Results

A total of 42 participants were enrolled in the study (12 PsA and/or PsO patients who were started on biologic agents, 18 PsO patients treated with non-biologic therapies, and 12 patients in the non-inflammatory control group with osteoarthritis). In the biologics group, 5 patients were started on an anti-TNF-α agent (4 adalimumab and 1 etanercept), 4 on an IL-17 inhibitor (secukinumab), and 2 on an IL-12/23 inhibitor (ustekinumab). One patient in the biologic group dropped out after having an abnormal imaging study that required further intervention, 5 patients in the PsO group not on biologic therapies withdrew after baseline imaging studies, and 2 patients in the control group withdrew after baseline imaging studies. This resulted in a total of 34 patients having complete baseline and follow-up imaging data for analysis: 11 PsA and/or PsO patients started on biologic agents, 13 PsO patients treated with non-biologic therapies, and 10 patients in the non-inflammatory control group with osteoarthritis. Additionally, all patients (including patients that withdrew/were lost to follow-up) were included in the sensitivity analysis with multiple imputation.

Participant characteristics are described in Table 1. In summary, 64.7% of participants were men, and the median age was 62 years (IQR: 48, 69), which was similar between the three groups. Median BMI was 28.7 kg·m^−2^ (IQR: 27.1, 36.3). More patients in Group 1 had diabetes (*P* = .032), were more commonly treated with Methotrexate (*P* = .002), or on medications, such as oral steroids (*P* < .001) and non-steroidal anti-inflammatory drugs (NSAIDs) (*P* = .002).

**Table 1:** Baseline characteristics of population

Variable	PsA and/or PsO on Biologics (n = 11)	PsO on non-biologics (n = 13)	Control (n = 10)	P value
	Median (IQR) or n (%)			
Age, years	62.0 (44.0, 64.5)	57.0 (47.5, 64.5)	63.5 (55.0, 73.0)	.218
Male sex, n (%)	5 (45%)	8 (62%)	7 (70%)	.525
BMI, kg·m^−2^	35.3 (28.7, 41.4)	29.6 (27.3, 33.6)	28.2 (25.0, 29.4)	.067
Hypertension, n (%)	5 (45%)	5 (38%)	1 (10%)	.186
Diabetes, n (%)	3 (27%)	0 (0%)	0 (0%)	.032**
Dyslipidemia, n (%)	3 (27%)	5 (38%)	4 (40%)	.793
Current smoking, n (%)	9 (91%)	8 (62%)	5 (50%)	.299
PASI of patients with PsO	(n = 2) 20.0 (10.4, 29.6)	7.0 (5.7, 10.2)	N/A	.132*
CRP, mg·L^−1^	4.10 (1.33, 4.98)	1.90 (.95, 5.38)	1.80 (1.10, 3.20)	.634
Other non-biologic medications/treatments				
NSAIDs, n (%)	5 (45%)	0 (0%)	0 (0%)	.002**
Steroids, n (%)	7 (64%)	0 (0%)	0 (0%)	.0001**
Methotrexate, n (%)	5 (45%)	0 (0%)	0 (0%)	.002**
Topical agents, n (%)	7 (64%)	10 (77%)	5 (50%)	.406
Acitretin, n (%)	1 (9%)	0 (0%)	0 (0%)	.341
Phototherapy, n (%)	2 (18%)	8 (62%)	0 (0%)	.004**

*BMI*, body mass index; *CRP*, c-reactive protein; *NSAIDs*, non-steroidal anti-inflammatory drugs; *PASI*, psoriasis area sensitivity index; *PsA*, psoriatic arthritis; *PsO*, cutaneous psoriasis; *TBR*, target-to-background ratio *Mann–Whitney test used to compare PASI values of groups **Statistically significant findings across groups on Kruskal–Wallis or Fisher exact testing

### Inflammation imaging results

**Figure 1:**
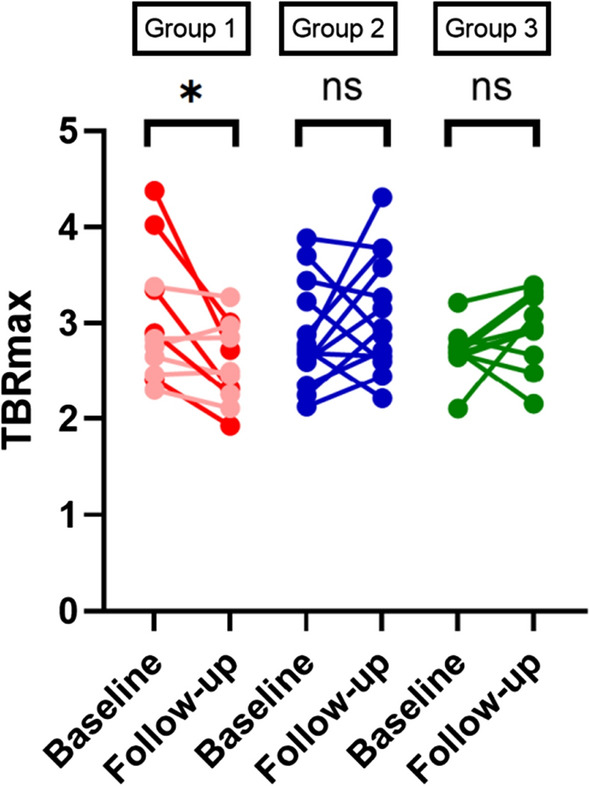
Change in ascending aorta FDG uptake from baseline to 6-month follow-up in Group 1 (patients with PsA and/or PsO on biologics), Group 2 (patients with PsO on non-biologics), and group 3 (control group). *FDG*, F-18-fluorodeoxyglucose; *PsA*, psoriatic arthritis; *PsO*, psoriatic disease; *TBR*, target-to-background ratio

**Figure 2:**
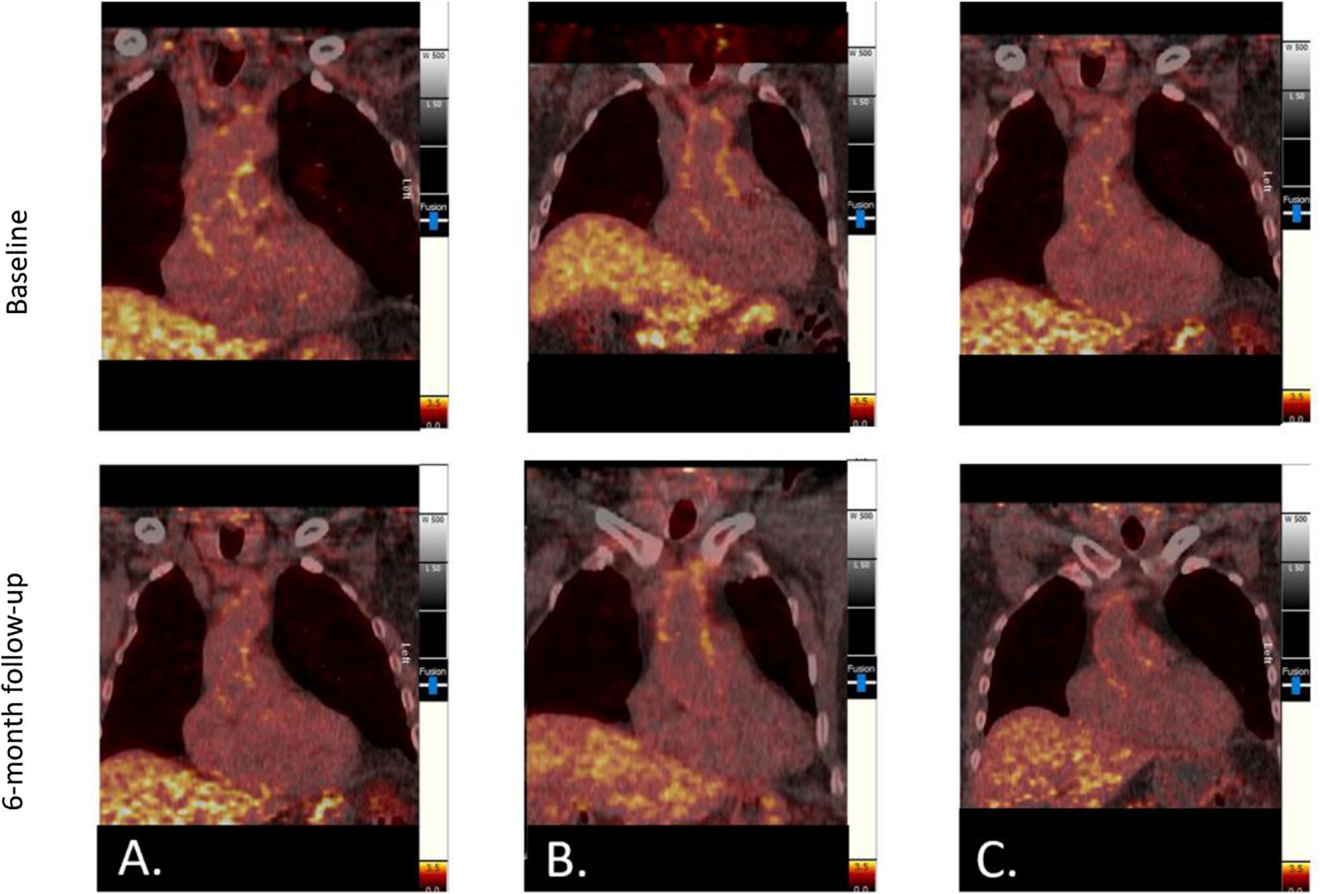
FDG images showing change in ascending aorta FDG uptake from baseline to 6-month follow-up in (**A**) patients with PsA and/or PsO on biologic agents, (**B**) patients with PsO on non-biologic agents, and (**C**) control patients with non-inflammatory arthritis. *FDG*, F-18-fluorodeoxyglucose; *PsA*, psoriatic arthritis; *PsO*, psoriatic disease

Baseline ascending aortic TBR_max_ was not statistically different between the three groups: 2.84 (IQR 2.51, 3.37), 2.73 (IQR 2.54, 3.28), versus 2.70 (IQR 2.68, 2.76) in the biologics, non-biologic, and control groups, respectively (*P* = .725). Similarly, baseline aortic arch TBR_max_ did not differ across the groups (*P* = .565) and nor did baseline descending aortic TBR_max_ (*P* = .190). For the primary outcome, analysis within groups with Wilcoxon Rank Sum testing demonstrated a statistically significant reduction in vascular inflammation measured as FDG TBR_max_ within the ascending aorta, in the biologic group only [baseline 2.84 (IQR 2.51, 3.37), follow-up 2.50 (IQR 2.27, 2.94) (*P* = .033)]. There were no significant changes in either the non-biologic [baseline 2.73 (IQR 2.54, 3.28), follow-up 2.95 (IQR 2.64, 3.63) (*P* = .279)] or control groups [baseline 2.70 (IQR 2.68, 2.76), follow-up 2.94 (IQR 2.67, 3.28) (*P* = .114)] (Table 2 and Fig. 1). Similarly, there were statistically significant decreases in measured FDG TBR_max_ within the aortic arch (*P* = .002) and descending aorta (*P* = .007) within the biologic group, but not the other groups (*P* > .05). Table 2 illustrates the change in aortic TBR uptake in the ascending aorta as well as change in MFR of the three groups. GLMM for repeated measures analysis revealed the change in FDG TBR_max_ over the study period in Group 1 differed significantly from Group 2 (*β* ± SE: .697 ± .245, *P* = .004) and Group 3 (*β* ± SE: .670 ± .259, *P* = .010). This was true for the aortic arch and descending aorta as well. Sensitivity analysis with multiple imputation for missing FDG TBR_max_ values showed the results remained significant after imputation (biologic group versus non-biologic group: *β* ± SE: .651 ± .230, *P* = .005); biologic group versus control group: *β* ± SE: .631 ± .261, *P* = .016). The change in FDG uptake from baseline to follow-up is shown in Fig. 2.

**Table 2:** Changes in TBR_max_ and LV MFR by treatment group during trial period

	PsA and/or PsO on biologics (n = 11)	PsO on non-biologics (n = 13)	Control (n = 10)	*P* value
Median (IQR) or n (%)				
Baseline ascending aorta TBR_max_	2.84 (2.51, 3.37)	2.73 (2.54, 3.28)	2.70 (2.68, 2.76)	.725
Follow-up ascending aorta TBR_max_	2.50 (2.27, 2.94)	2.95 (2.64, 3.63)	2.94 (2.67, 3.28)	.102
Delta ascending aorta TBR_max_	− .19 (− .92, .11)*^,^***	.32 (− .26, .75)*	.21 (− .18, .58)*	.021**
Baseline ascending aorta SUV_max_	3.51 (3.08, 4.16)	3.63 (3.02, 4.72)	3.61 (3.00, 3.96)	.803
Follow-up ascending aorta SUV_max_	3.26 (2.67, 3.47)	3.86 (3.02, 4.37)	3.51 (3.33, 3.82)	.102
Delta ascending aorta SUV_max_	− .42 (− .83, − .07)***	.23 (− .62, .57)	.08 (− .61, .74)	.103
Baseline aortic arch TBR_max_	2.76 (2.66, 3.06)	2.62 (2.43, 3.28)	2.87 (2.68, 3.12)	.565
Follow-up aortic arch TBR_max_	2.47 (2.12, 2.85)	2.89 (2.51, 3.19)	2.97 (2.44, 3.24)	.079
Delta aortic arch TBR_max_	− .43 (− .63, − .29)*^,^***	.16 (− .47, .52)*	.20 (− .31, .48)*	.029**
Baseline aortic arch SUV_max_	3.60 (3.07, 4.08)	3.13 (2.79, 4.10)	3.59 (3.54, 4.15)	.640
Follow-up aortic arch SUV_max_	3.04 (2.46, 3.33)	3.57 (3.05, 4.01)	3.50 (3.10, 4.12)	.068
Delta aortic arch SUV_max_	− .53 (− .91, − .24)***	.29 (− .17, .73)	− .31 (− .46, .46)	.045**
Baseline descending aorta TBR_max_	2.73 (2.47, 3.46)	2.71 (2.39, 3.14)	3.25 (2.84, 3.35)	.190
Follow-up descending aorta TBR_max_	2.25 (2.01, 2.54)	2.78 (2.59, 3.15)	3.22 (2.77, 3.66)	.002**
Delta descending aorta TBR_max_	− 57 (− .79, − 0.17)*^,^***	.07 (− .24, .24)*	.12 (− .47, .50)*	.011**
Baseline descending aorta SUV_max_	3.70 (2.83, 3.98)	3.41 (3.01, 3.80)	3.82 (3.66, 4.33)	.099
Follow-up descending aorta SUV_max_	2.85 (2.46, 3.04)	3.46 (3.20, 4.00)	3.64 (3.34, 3.96)	.002**
Delta descending aorta SUV_max_	− .74 (− .95, − .27)***	.20 (− .18, .52)	− .29 (− .50, .02)	.012**
Flow data				
Baseline LV MFR	2.96 (2.38, 3.46)	3.14 (2.54, 3.95)	3.43 (2.84, 4.34)	.528
Follow-up LV MFR	2.85 (2.20, 3.06)	3.58 (3.08, 3.94)	3.92 (2.97, 4.38)	.035**
Delta LV MFR	− .24 (− .46, − .02)***	.13 (− .38, .73)	.30 (− .83, .96)	.246
Baseline rest SBP	127 (104, 140)	134 (117, 142)	110 (102, 127)	.098
Baseline rest HR	68 (58, 75)	72 (66, 82)	63 (55, 67)	.023**
Baseline rest flow	.80 (.58, .80)	.89 (.72, 1.0)	.62 (.52, .71)	.061
Baseline stress flow	1.79 (1.64, 2.71)	2.66 (2.40, 2.92)	2.12 (1.89, 2.62)	.127
Baseline stress SBP	135 (123, 141)	141 (125, 145)	117 (110, 140)	.049***
Baseline stress HR	90 (83, 95)	96 (87, 102)	80 (77, 87)	.020***
Follow-up rest SBP	133 (111, 145)	123 (117, 140)	117 (102, 135)	.400
Follow-up rest HR	67 (63, 79)	67 (64, 73)	60 (58, 61)	.003***
Follow-up rest flow	.79 (.67, 1.01)	.74 (.66, .87)	.66 (.55, .88)	.200
Follow-up stress SBP	142 (126, 159)	130 (124, 141)	118 (107, 144)	.172
Follow-up stress HR	85 (76, 95)	93 (87, 97)	83 (75, 88)	.067
Follow-up stress flow	2.15 (1.60, 3.08)	2.60 (2.28, 2.94)	2.36 (2.20, 2.72)	.528

*HT*, heart rate; *LV MFR*, left ventricular myocardial blood flow reserve; *MFR*, myocardial blood flow reserve; *PsA*, psoriatic arthritis; *PsO*, cutaneous arthritis; *SBP*, systolic blood pressure; *TBR*, target-to-background ratio

*Statistically significant findings on GLMM for repeated measures analysis revealed the change in FDG TBRmax over the study period in Group 1 differed significantly (*P* < .05)

**Statistically significant findings across groups on Kruskal–Wallis testing.

***Within-group testing of changes in baseline and 6-month values were statistically significant with Wilcoxon Signed Rank testing

Regarding the exploratory outcome, baseline MFR was not statistically different between the three groups: Baseline MFR was 2.96 (IQR 2.38, 3.46), 3.14 (IQR 2.54, 3.95), and 3.43 (IQR 2.84, 4.34) in the biologic, non-biologic, and control groups, respectively (*P* = .528). Analysis within groups with Wilcoxon Rank Sum testing demonstrated a statistically significant reduction in MFR in the biologic group only [baseline 2.96 (IQR 2.38, 3.46), follow-up 2.85 (IQR 2.20, 3.06) (*P* = .037)]. There were no significant changes in either the non-biologic [baseline: 3.14 (IQR 2.54, 3.95), follow-up: 3.58 (IQR 3.08, 3.94) (*P* = .279)] or control groups [baseline: 3.43 (IQR 2.84, 4.34), follow-up: 3.92 (IQR 2.97, 4.38) (*P* = .114)] (Table 2). GLMM for repeated measures analysis revealed no statistically significant differences in the change in MFR over the study period between the biologic group and non-biologic group (*β* ± SE: .477 ± .370, *P* = .198) and control group (*β* ± SE: .467 ± .395, *P* = .237), respectively.

Sensitivity analysis with multiple imputation for missing ascending aortic FDG TBR_max_ values showed that results remained non-significant even after imputation (biologic group versus non-biologic group: *β* ± SE: .522 ± .407, *P* = .201; biologic group versus control group: *β* ± SE: .384 ± .456, *P* = .400).

**Figure 3:**
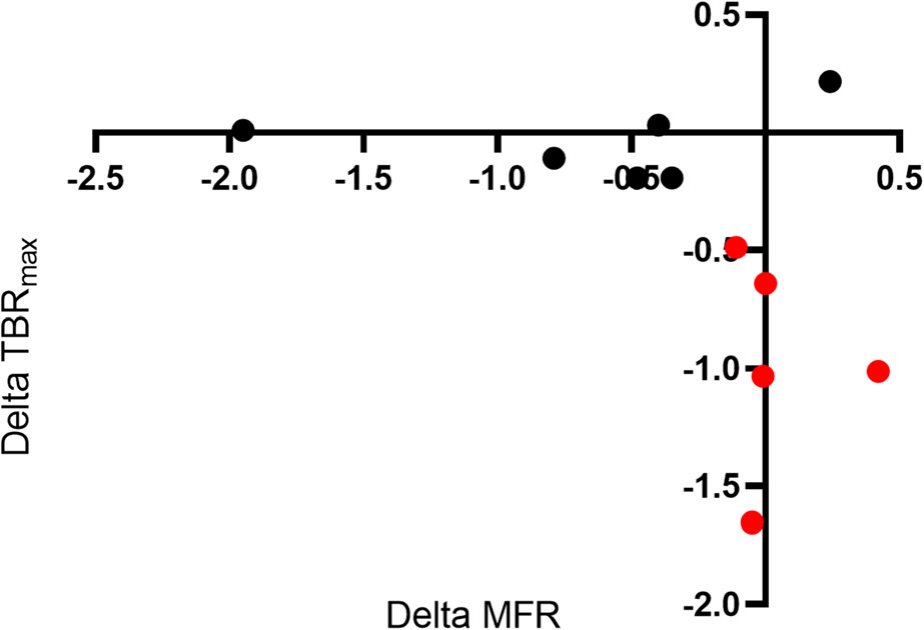
Correlation between change in TBR_max_ and MFR in patients with PsA and/or PsO on biologic agents. Patients with a clinically significant response to biologic agents are colored red. *Delta TBR*_*max*_, target-to-background ratio in the most diseased segment of the ascending aorta at baseline compared to 6-month follow-up; *Delta MFR*, myocardial blood flow reserve at baseline compared to 6-month follow-up

While GLMM for repeated measures analysis revealed no significant difference in the change in MFR between the three groups over the study period, to better understand the relationship of changes in vascular inflammation to microvascular function, we decided to further evaluate the change in MFR in the subgroup of PsA patients treated with biologic agents in a post hoc analysis. We compared the MFR of patients in the biologic group who had a response in their TBR_max_ that was greater or equal to the median of this group to those who had a response that was less than the median. We found a significant difference in these groups of patients. The group with a clinical response to biologic agents as measured by a change in TBR_max_ that was greater or equal to the median change (7%) maintained MFR (3.40 ± 1.23 MFR to 3.5 ± 1.2 MFR over 6 months) when compared to the group with a below median response on TBR_max_ which had a drop in MFR (2.9 ± .8 MFR to 2.2 ± .6 over 6 months; *P* = .03). Spearman’s coefficient of rank correlation (rho) between change in TBR_max_ and change in MFR was .709 (*P* = .0146) (Fig. 3). Finally, as an additional exploratory analysis we looked at whether there were any differences in vascular inflammation change between patients in Group 1 on different types of biologics (TNF inhibitor, IL-17 inhibitor, and IL-12/23 inhibitor). We found no difference, likely due to the small number of patients.

## Discussion

We conducted a prospective cohort study to determine the impact of systemic anti-cytokine treatment on vascular inflammation and MFR in patients with psoriasis, an inflammatory disease that is well known to be associated with vascular inflammation.^8^ In a cohort of psoriasis patients that were started on biologic therapy, representative of clinical practice, we found that FDG uptake in the thoracic aorta decreased over the 6-month study period compared to no change in psoriasis patients treated with non-systemic therapies or in a cohort of control patients with non-inflammatory joint and skin disease. Additionally, we found that in those psoriasis patients treated with biologics who had imaging evidence of a significant anti-inflammatory response to the biologics, MFR was maintained compared to the group that did not have a significant response to the biologic therapy. The present study helps fill a knowledge gap by suggesting that anti-cytokine therapy may have vascular anti-inflammatory effects. These changes may be linked to changes in the coronary microvasculature in patients with psoriasis and support the notion that anti-inflammatory biologics may have a role in mitigating CV disease in this patient population.

Psoriasis disease is a chronic inflammatory skin condition that affects over 125 million people worldwide.^27^ Patients with psoriasis have been found to have an increased incidence of CV disease and increased risk of MACE.^3,28–30^ A key link between psoriasis and heart disease is mediated through inflammation. Patients with psoriasis have increased vascular inflammation measured by FDG PET, and it has been shown that increasing severity of skin disease is associated with increased vascular inflammation.^8,31^ However, at this point it is not yet known whether targeting inflammatory pathways could lead to downstream effects in reducing CV morbidity and mortality in these patients.

In our jurisdiction, PsA patients progressing to a biologic agent need to have ongoing arthritis (5 or more swollen joints) despite treatment with methotrexate for 3 months (usually at doses between 20 and 25 mg·week^−1^) and an additional 3 months on leflunomide (20 mg·day^−1^) or sulfasalazine (2000 mg·day^−1^). Patients with skin disease need to demonstrate at least 3% of total body skin involvement despite 3 months of treatment with systemic therapy, usually methotrexate or cyclosporine. In the present study, treatment with anti-cytokine therapy was associated with a clinically significant reduction in aortic inflammation both when compared within the biologic group and also when compared to the non-biologic and control groups. Our data provide insights into the potential linkage of atherosclerosis with inflammation and further suggests that treatment of psoriasis patients with biologic agents may help to mitigate vascular inflammation. Additionally, while the overall change in MFR did not differ between the psoriasis group treated with anti-cytokine therapy and the other groups, when we looked specifically at patients in the psoriasis group that were treated with biologic agents who had a response in their TBR_max_ that was greater than the median (compared to the patients treated with biologic agents with a response less than the median), MFR was maintained in the responders compared to the non-responders (where it significantly dropped).

Our results are consistent with a noncontrolled study in rheumatoid arthritis of anti-TNF-α therapy, which demonstrated a reduction in vascular inflammation on FDG PET imaging after 8 weeks of treatment.^32^ They are also consistent with an observational cohort from Kim et al. evaluating the anti-inflammatory effect of 25 patients with PsO treated with Ustekinumab.^33^ Furthermore, an observational study from Dey et al. that evaluated a cohort of psoriasis patients being treated systemically had similar findings.^34^

Our results, however, are not consistent with a randomized placebo-controlled trial from Mehta et al., which showed no change in vascular inflammation on FDG PET in patients randomized to adalimumab, phototherapy, or placebo for a period of 12 weeks, with an open-label extension of the adalimumab arm for a period of 52 weeks.^35^ There are several possible reasons for the discrepancy of our results with that observed by Mehta et al. Firstly, our study had 64% of the PsA/PsO population with PsA (while only 10% in the Mehta et al., study had PsA). Thus, there are underlying differences in the composition of the study populations which could theoretically influence the vascular response to biologic therapy. Secondly, while there was no difference in baseline inflammatory levels between our three study groups, it should be noted that our biologic therapy group had a higher proportion of patients pretreated with NSAIDS, methotrexate, and steroids. This difference in anti-inflammatory pretreatment across study groups could again theoretically alter the vascular response to biologic agents. Thirdly, our sample size was very small, increasing the risk for our results to be influenced by statistical chance. However, the consistent response of different anatomical sections of the aorta to the biologic agents certainly argues against this explanation. Lastly, ours was a non-randomized study, which could result in potential unmeasured confounding of our results. It should be noted, however, that these possible explanations are purely speculative.

Our study has several important limitations. Firstly, this was a non-randomized prospective observational study with a modest sample size, and limited clinical follow-up. Thus, our results should be interpreted with caution, and while they are largely hypothesis generating, they should certainly fuel further randomized clinical trials to investigate these findings. Conversely, we employed rigorous methodological analyses as well as a sensitivity analysis with multiple imputation to attempt to overcome some of these limitations and strengthen our results. Secondly, we utilized imaging outcomes to measure inflammatory response to biologic therapy rather than clinical outcomes. While clinical assessment of joint response to biologic therapy involves subjective measures, the imaging data of vascular inflammation that we utilized for our primary outcome are a completely objective measure of treatment response. Both vascular inflammation and MFR have been shown to be robust indices, which can give important insights into disease activity over shorter periods of clinical observation. Therefore, both vascular inflammation and MFR represent important surrogate markers for imaging trials that allow for the assessment of therapeutic benefit of interventions in a timely manner. In this regard, while FDG PET has been evaluated utilized extensively for the non-invasive detection of inflammation related to atherosclerosis, it does have several limitations which should be highlighted. While FDG PET imaging targets activated macrophages, it is still a non-specific probe that can accumulate in other metabolically active tissues which can therefore introduce interfering background signal.^36,37^ Imaging of the ascending aorta requires strict dietary preparation to suppress FDG-myocardium uptake (with a high-fat, low-carbohydrate diet prior to PET imaging), given the significant background myocardial FDG uptake which can make it more difficult to differentiate vascular inflammation from background uptake.^38,39^ FDG PET imaging is also affected by glucose levels, so particular caution in imaging patients with diabetes and hyperglycemia must be employed.^40^ From a technical perspective, the partial volume effect and motion artifacts from cardiac and organ motion during imaging all limit the spatial resolution and specificity of the test.^41^ Additionally, given the limited spatial resolution of current PET imaging systems, we are currently unable to directly quantify “vulnerable plaque” in smaller-sized vessels, such as the coronary arteries meaning we are limited to assessing larger caliber vessels, such as the aorta.^42^ Finally, while baseline demographic factors were not statistically different between groups, it is possible that the numeric difference in the proportion of participants with cardiac risk factors like hypertension, diabetes or current smoking, or differences in baseline BMI could have influenced the differences we saw in baseline TBR_max_ and response to biologics between the groups.

In conclusion, our study suggests that anti-cytokine therapy is associated with decreasing vascular inflammation in patients with PsA as assessed by FDG PET in contrast to non-biologics as well as a non-inflammatory control group. While our study should be interpreted with caution given the small number of participants and limited generalizability, our results do suggest an association between these biologic agents and preserved MFR in patients who respond favorably in terms of their vascular inflammation. This study supports the notion that there is a positive impact of immune therapies on vascular inflammation and microvascular disease in patients with chronic inflammation; however, larger prospective clinical outcome trials investigating this are warranted.

## New knowledge gained

Anti-cytokine therapy is associated with decreasing vascular inflammation in patients with psoriatic arthritis as assessed by FDG PET.

## Data Availability

Data is available upon request.
